# Handoff Communication between Remote Healthcare Facilities

**DOI:** 10.1097/pq9.0000000000000269

**Published:** 2020-03-20

**Authors:** Sara Helmig, Jennifer Cox, Brinda Mehta, Jonathan Burlison, Jennifer Morgan, Carolyn Russo

**Affiliations:** From the * Department of Oncology, St. Jude Children’s Research Hospital, Memphis, Tenn.; † Department of Pediatrics, St. Jude Affiliate Clinic at Huntsville Hospital for Women and Children, Huntsville, Ala.; ‡ Department of Pediatrics, St. Jude Affiliate Clinic at Children’s Hospital of Illinois, Peoria, Ill.; § Department of Quality and Safety, St. Jude Children’s Research Hospital, Memphis, Tenn.; ¶ Affiliate Program Office, St. Jude Children’s Research Hospital, St. Jude Children’s Research Hospital, Memphis, Tenn. Department of Hematology, St. Jude Children’s Research Hospital, St. Jude Children’s Research Hospital, Memphis, Tenn; ‖ Department of Hematology, St. Jude Children’s Research Hospital, St. Jude Children’s Research Hospital, Memphis, Tenn.

## Abstract

**Introduction:**

Handoffs and transitions of care are common weak points in healthcare provider communication as patients move between sites. With no consistent pattern of communication between St. Jude Children’s Research Hospital (St. Jude) and its affiliated clinics, the Affiliate Program Office at St. Jude developed and implemented a standardized communication tool to facilitate patient transitions between different healthcare sites.

**Methods:**

Each team of providers created flow diagrams to define the current state of communication when patients were transitioning between remote sites. Fishbone diagrams identified the common barriers to effective communication as a lack of consistent communication and ownership. We developed a communication tool to address these barriers, which was disseminated by secure email. We measured the percent usage of the completed hand-off tool before a patient transitioned, staff experience, and the number of errors.

**Results:**

The time to send or receive the communication bundle was <10 minutes. Within 3 months of implementing the SMART bundle at 3 pilot sites, the bundle was used completely in 6 of 8 patient transitions and was associated with somewhat improved staff satisfaction. We identified no adverse events related to the communication bundle.

**Conclusions:**

In this small pilot study, we accomplished closed-loop communication between geographically remote healthcare sites by using an electronically transmitted standardized communication bundle.

## INTRODUCTION

According to recent data reported by The Joint Commission, failures in communication are a contributing root cause for approximately 80% of sentinel events.^1^ Transitions of patient care between medical teams are prone to miscommunication.^2–4^ With duty hour changes, resident handoff communication is a noted weakness, resulting in safety concerns.^5^ Several collaborative groups have improved resident handoff communication by applying a structured approach to information exchange in the form of a mnemonic.^3,4^ A consistent pattern of communication creates a shared mental model between the deliverer and recipient, in which communication gaps are noted and corrected in person and in real time. Most notably, the I-PASS Study Group implemented a structured, highly reliable communication tool for resident physicians termed I-PASS (ie, *i*llness severity, *p*atient summary, *a*ction list, *s*ituation awareness and contingency planning, and *s*ynthesis by the receiver).^6,7^ After implementing I-PASS handoffs, medical errors decreased by 23%, and preventable adverse events decreased by 30%.^8^ However, such handoff communication interventions have involved resident or nurse handoffs and transfers within a single physical location, with direct person-to-person interactions.^1,9^

When the transition of care is extended to different facilities without face-to-face interactions, a breakdown in patient care communication is commonplace, and a source of preventable adverse events.^9,10^ Failures in communication within the network of pediatric oncology clinics at St. Jude Children’s Research Hospital (St. Jude) led to inefficiencies in care. One example is a patient who required a specialized diagnostic imaging procedure at St. Jude; however, the affiliate site failed to communicate when the patient would return from the affiliate site to St. Jude, and the procedure was not scheduled.

A second example is a patient who returned to the affiliate site from St. Jude to receive chemotherapy; however, the St. Jude team failed to communicate the message to the affiliate team, and the chemotherapy administration was delayed. Both these situations caused patient dissatisfaction. We must ensure that patient information exchange is efficient and complete and benefits patient safety and trust.

With no consistent pattern of communication between the St. Jude and its network of affiliated clinics, the Affiliate Program Office at St. Jude developed and implemented a standardized communication tool for patient transitions between different healthcare sites.

## METHODS

### Context

St. Jude Children’s Research Hospital (St. Jude) is a 78-bed pediatric hospital with integrated outpatient clinics offering subspecialty and surgical services for children with cancer, blood disorders, and other catastrophic diseases. Annually, it has ~3,500 annual inpatient admissions and sees ~7,500 patients, most requiring ongoing treatment in inpatient and outpatient settings for complex medical diagnoses. The Affiliate Program at St. Jude allows more children access to pediatric oncology care closer to patient homes. Currently, 8 clinics, located throughout the Southeast and the Midwest, are affiliated with St. Jude and contribute 35% of the patients enrolled in St. Jude clinical trials. This project focused specifically on children with solid tumors in 2 of the 8 clinics. Pediatric oncology patients with solid tumors may receive treatment at St. Jude in Memphis, Tenn., or an affiliated clinic. Depending on the type of treatment required, patients may travel at varying intervals between Memphis and an affiliated clinic with different clinical staff at each site, sharing the responsibility of care for such “shared patients.” When patients with solid tumors receiving active therapy arrive at their current treatment site, the providers may or may not be specifically aware of what occurred during previous visits at other sites or if any changes were made to patient treatment plans. Patients had a primary care team at each site, consisting of a pediatric oncologist, an advanced practice provider, and a primary nurse. Each site used different electronic medical record (EMR) systems that were not integrated. Affiliate clinic staff had remote electronic access to the St. Jude EMR, but this was not true for St. Jude staff accessing the affiliate’s EMRs. The St. Jude Institutional Review Board approved this quality improvement project.

### Intervention

**Fig. 1.**
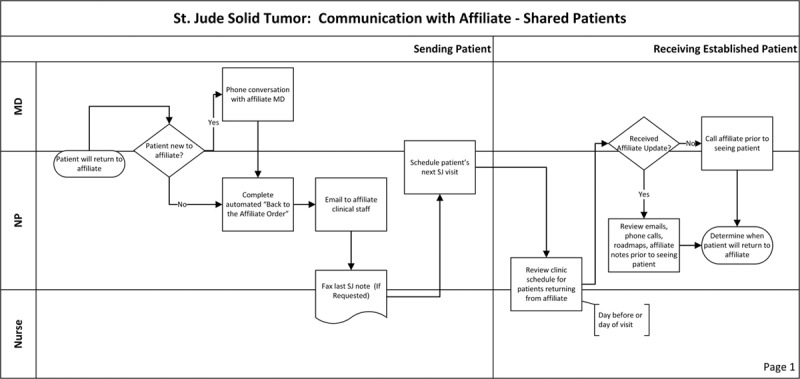
Process map of the initial state of communication created by the St. Jude Solid Tumor team. The centerline represents the patient transition between sites. To the left of the centerline described the process when the St. Jude Solid Tumor team sent a patient to an affiliate. To the right of the centerline described the process when the St. Jude Solid Tumor team received a patient from an affiliate. MD, physician; NP, advanced practice provider; SJ, St. Jude.

**Fig. 2.**
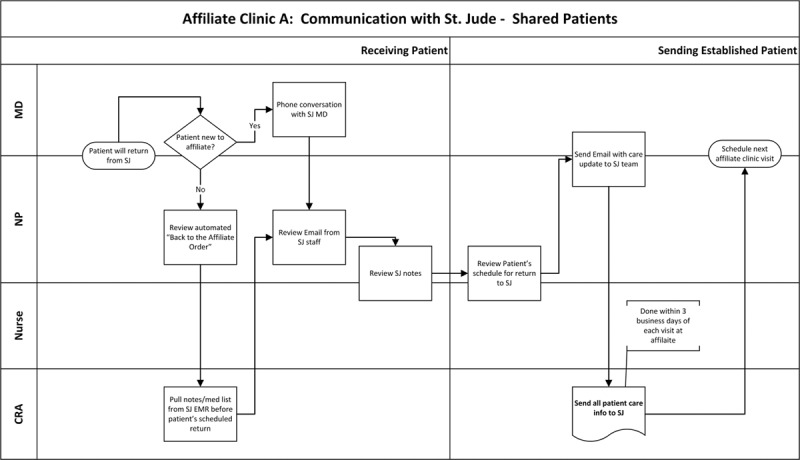
Process map of the initial state of communication created by Affiliate A. The centerline represents the patient transition between sites. To the left of the centerline described the process when the Affiliate A team sent a patient to the St. Jude Solid Tumor team. To the right of the centerline described the process when the Affiliate A team received a patient from the St. Jude Solid Tumor team. CRA, clinical research associate; EMR, electronic medical record; MD, physician; NP, advanced practice provider; ; SJ, St. Jude.

**Fig. 3.**
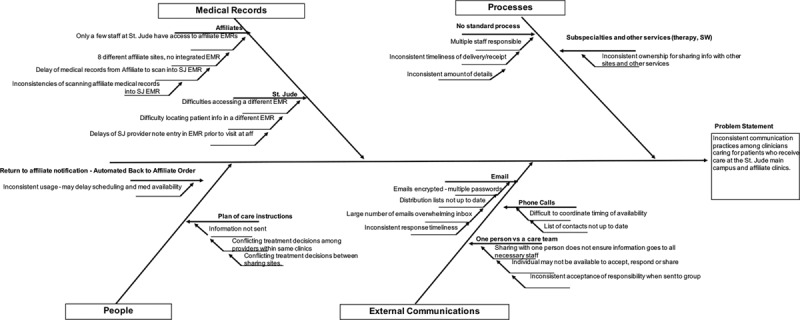
Cause and effect diagram created from the process maps. EMR, electronic medical record; SJ, St. Jude; SW, social worker.

**Fig. 4.**
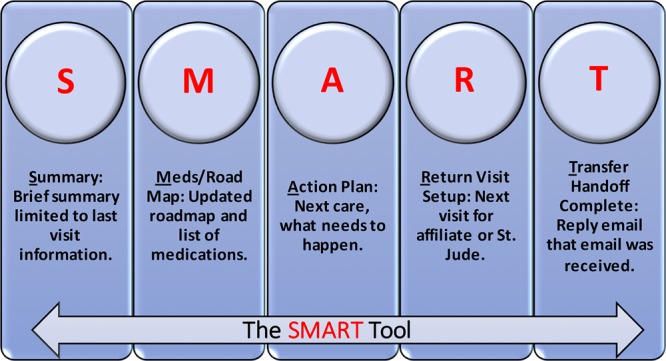
The SMART communication tool mnemonic.

This project began by each primary team at 3 sites (ie, St. Jude, Affiliate A, and Affiliate B) creating process flow maps that defined the current state of communication when patients transitioned between remote sites (Figs. 1 and 2). We selected only 2 affiliate sites for this pilot study. We started with the St. Jude Solid Tumor team, given the diverse tumor types were associated with a larger variety of patient transitions. The project leaders at each of the 3 sites facilitated this portion of the project. Each team identified their specific barriers to effective communication by using fishbone diagrams exploring cause and effect (Fig. 3). We identified common barriers of incomplete communication and the lack of closed-loop communication. We found that key details were not always included in communication between sites. In some cases, the information went to one person who may have been out of office. In a few cases, communication with the site was overlooked. Developing a structured, closed-loop communication tool delivered by email was considered as a potential method to improve communication. Using a modification of the I-PASS methodology, the team (coauthors) created an email communication tool termed SMART (mnemonic coined by the first author) (ie, a *s*ummary of the plan, *m*edications/roadmap, *a*ction plan, *r*eturn visit date, and *t*ransfer confirmation). The *receiver replies by confirming an* understanding of the care plan (Fig. 4). The communication tool was transmitted by secure email. The clinician who saw the patient at the visit before the transition was responsible for sending the SMART communication tool. Each team had an email distribution that included the primary care teams to receive the SMART communication tool. To ensure sustainability, we assigned SMART champions at each site who were tasked to disseminate the information and encouraged feedback. We sent email reminders every few weeks that included any positive feedback from staff.

### Study of the Intervention

This intervention aimed to achieve 80% SMART communication tool completion by the sending sites within 48 hours before patient arrivals at receiving sites within 3 months of implementing the intervention. The tool was sent via email by the providers who provided care for patients before their transition. They sent these emails to a distribution list of the receiving clinical teams. Email confirmation by a designated provider of the receiving team closed the loop. After the first 3-month period, we collected an additional 4 months of data.

### Measures

Process and outcome measures included the usage of the completed SMART tool before the transition, staff satisfaction, and the number of adverse events related to communication. Usage of the SMART tool was tracked over 3 months (March 2019–May 2019) each time a patient with a solid tumor transitioned between Memphis and Affiliate A or Affiliate B in either direction. The Affiliate Nurse Director was included in the email distribution lists to track the process measure. We measured the number of times the SMART communication tool and the date/time the tools were used when the patient transitioned. Through the St. Jude EMR system, the time and date when a patient transitioned between sites were tracked.

The staff at each site was surveyed about the SMART process at monthly teleconferences during the 3 months. An annual communication survey started before this project was used to assess overall satisfaction with communication before and after the project. A web-based survey was sent to providers at both the affiliate sites and the providers at St. Jude. The communication survey inquired about effective communication with a Likert scale of 1–5: 1 = very ineffective; 2 = ineffective; 3 = neutral; 4 = effective; and 5 = very effective communication. Adverse events related to the communication tool were collected monthly on a nursing dashboard, an online self-survey hosting site that measured medication variances, treatment deviations, central line problems, specimen variances, transfusion variances, device or equipment failures, sentinel event, and any other patient care event.

### Analysis

The ratio of the number of times the SMART tool was used versus the total number of transitions of patients with solid tumors between the affiliate sites and the St. Jude campus was determined. We only counted compliance with the process if the sender used the SMART tool, and the receiver confirmed receipt of the email. Because this project was a new process, we could not make comparisons to a baseline measure. Staff satisfaction was qualitatively measured with a mean postsurvey score. Also, an annual survey on communication, which started before this project, provided a “before and after” comparison. Adverse events recorded on the monthly dashboard were tallied. Data were reviewed monthly by the Affiliate Nursing Director and shared with the team during monthly conference calls.

## RESULTS

**Fig. 5.**
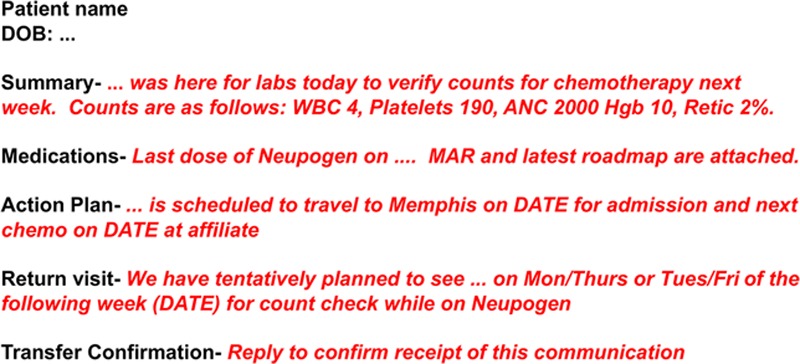
Example of a SMART email from the Affiliate A team to the St. Jude Solid Tumor team with the patient name and other identifying information were redacted to protect privacy. ANC, absolute neutrophil count; hgb, hemoglobin; MAR, medical administration record; retic, reticulocyte count; WBC, white blood count.

Receivers and senders said that the SMART tool generally required 5–10 minutes to compose and read. The receiver replied to the sender confirmed the receipt. A representative SMART email with patient information redacted is shown in Figure 5.

The SMART bundle was used with 75% of solid tumor patient transitions during the 3 months. There were 8 patient transitions, of which 6 transitions used the SMART bundle with a confirmed receipt by the receiver. In the following 4-month period, there were 4 patient transitions, and each transition used the SMART bundle, increasing the percent usage to 83%. Staff at all 3 sites reported ease of use and appreciated the concise patient information. A self-report survey evaluating affiliate provider perceptions of effective communication was administered pre and post SMART implementation. Communication was evaluated via anchored Likert-scaled responses as described in the Methods. The preimplementation and postimplementation results were not normally distributed. The preimplementation response rate was 40% (18 of 45), and the median response was 3.5. The postimplementation response rate was 46% (21 of 45), and the median response was 3.5. The results of a Mann–Whitney U test comparing the 2 groups was not significant (*U = 616*.0, *P* = 0.12). Although this was not statistically significant, comments from the survey were positive. A few examples from the receivers are as follows: “I love the SMART form we’ve been using. Getting it ingrained in our emails makes things much better.” “The structured email template is great.” “These emails have been a great help to patient care.” We noted no harm or adverse events related to the communication tool was identified during the 7 months.

## DISCUSSION

An in-person structured handoff tool was adapted and piloted in a small sample size to facilitate electronic patient transition communication between distant healthcare facilities. Within 3 months of implementation, usage of the handoff tool was accomplished in 6 of 8 transitions. Although numeric survey scores did not change, the providers reported improved communication because of the intervention.

Improving handoff communication is an important goal for medical teams and has been the subject of many healthcare publications over the past 2 decades. Optimal handoff communication has become a more pronounced concern with reductions in resident work hours. Many recent studies have focused on trainee handoffs within one physical system or from one service to another service within a single hospital.^11,12^ Each clinical setting presents distinct challenges. An important difference between most previously published studies and the quality improvement project reported here is that the patient handoffs occurred between geographically distant sites, with cancer care delivered at both sites. The St. Jude Affiliate Program structure may be unique; however, as healthcare evolves with the centralization of specialized care in major centers and delivery of care in the patients’ local communities, this type of patient care transition between providers at the main campus and those in remote sites will most likely become more common.

Communication is complex, comprising language and context. A proficient fictional writer uses language to transmit context to readers, but current EMR systems do a poor job in transmitting the shared mental models needed for high-functioning healthcare teams.^13^ A shared mental model is often best delivered in person, with direct eye contact, appreciation of body language, and verbal cues, so that the receiver understands what the sender is communicating exactly.^9^

Virtual communication is essential in many fields today, including healthcare. When providers are positioned in different geographic locations with different time zones, virtual communication can suffer without a shared understanding of the tasks to be performed.^14^ Email is unidimensional and lacks a shared mental model. Moreover, email communication risks potential time delays. One method to overcome this barrier is to have structured communication delivered within a prescribed time frame, as illustrated in this report. Using a structured template can avoid key content omissions. Another key component to enhancing virtual communication is for the receiver to confirm receipt to the sender, which was an important component of the SMART tool. Closing the loop of communication added to the coordination of care. It confirmed to the sender that the message was received.

Involving both senders and receivers in developing and implementing the communication tool was critical to its success. As others have shown, a disconnect may exist between the perceptions of the sender and the receiver. Berendsen et al^15^ found highly significant (*P* < 0.001) disagreement in the perceptions of written communication between specialists at a main campus and practitioners at remote sites. Specialists perceived their communication to be timely and complete, whereas the practitioners in the remote sites did not. In contrast, the practitioners considered their availability and communication to be adequate, whereas the specialists at the main site did not. Understanding the process flow of communication from both sides builds trust and aids more effective communication.

Effective communication can not only positively influence patient safety and satisfaction but also reduce frustration among healthcare staff.^16^ We did not measure the effect of provider burnout in this study; however, others have documented that poor communication leads to additional workload or duplication of effort, which most likely exacerbates provider burnout. Of all the interventions to address provider burnout, improving effective communication between providers may be a relatively high-impact, low-resource method to reduce burnout.

Several limitations to this work should be noted. This mode of structured email communication is not appropriate for patient emergencies. For urgent issues or complex social situations, telephone calls may be necessary. Direct conversations are always the most effective method of communication. Usage of the tool was not 100%, and inconsistent usage or lack of response to the email tool defeats the purpose of effective communication.

A major limitation of this project is the small sample size. This pilot project included only 2 of 8 affiliated clinics and focused only on patients with solid tumors intending to develop a hand-off tool rather than to validate the hand-off tool. However, given the initial results, we plan to extend the communication bundle to all affiliate sites and all clinical services, which will allow for analysis with a larger sample size. The goal is to have the hand-off tool completed with every patient transition.

## CONCLUSIONS

In this pilot study, closed-loop, high-reliability communication was accomplished between geographically remote healthcare sites by using a standardized communication tool that is transmitted electronically.

## DISCLOSURE

The authors have no financial interest to declare in relation to the content of this article.

## ACKNOWLEDGMENTS

St. Jude Children’s Research Hospital contracted with the I-PASS Institute to provide guidance and tools for best practices to implement and adapt the I-PASS handoff program to our hospital. The authors specifically acknowledge Dan West, MD, Amy Starmer, MD, William Floyd, and Christina Hazekamp from the I-PASS Institute. The I-PASS Institute had no role in preparing this manuscript. The authors thank Nisha Badders, Ph.D., ELS, for the scientific editing of the manuscript. The authors also thank the clinic staff for their participation in the project and continuing to drive to improve patient care.
